# The Eternal Robot: Anchoring Effects in Humans' Mental Models of Robots and Their Self

**DOI:** 10.3389/frobt.2020.546724

**Published:** 2020-12-18

**Authors:** Daniel Ullrich, Andreas Butz, Sarah Diefenbach

**Affiliations:** ^1^Department of Computer Science, Ludwig-Maximilians-University Munich, Munich, Germany; ^2^Department of Psychology, Ludwig-Maximilians-University Munich, Munich, Germany

**Keywords:** human-robot-interaction, mental models, human-likeness, robotness, anchoring effects, design goals

## Abstract

Current robot designs often reflect an anthropomorphic approach, apparently aiming to convince users through an ideal system, being most similar or even on par with humans. The present paper challenges human-likeness as a design goal and questions whether simulating human appearance and performance adequately fits into how humans think about robots in a conceptual sense, i.e., human's mental models of robots and their self. Independent of the technical possibilities and limitations, our paper explores robots' attributed potential to become human-like by means of a thought experiment. Four hundred eighty-one participants were confronted with fictional transitions from human-to-robot and robot-to-human, consisting of 20 subsequent steps. In each step, one part or area of the human (e.g., brain, legs) was replaced with robotic parts providing equal functionalities and vice versa. After each step, the participants rated the remaining humanness and remaining self of the depicted entity on a scale from 0 to 100%. It showed that the starting category (e.g., human, robot) serves as an anchor for all former judgments and can hardly be overcome. Even if all body parts had been exchanged, a former robot was not perceived as totally human-like and a former human not as totally robot-like. Moreover, humanness appeared as a more sensible and easier denied attribute than robotness, i.e., after the objectively same transition and exchange of the same parts, the former human was attributed less humanness and self left compared to the former robot's robotness and self left. The participants' qualitative statements about why the robot has not become human-like, often concerned the (unnatural) process of production, or simply argued that no matter how many parts are exchanged, the individual keeps its original entity. Based on such findings, we suggest that instead of designing most human-like robots in order to reach acceptance, it might be more promising to understand robots as an own “species” and underline their specific characteristics and benefits. Limitations of the present study and implications for future HRI research and practice are discussed.

## Introduction

Current robot designs often reflect an anthropomorphic approach, aiming at human-like visual appearance or simulating human communication behavior. While in principle, robot designs can be of many different types and morphologies (e.g., humanoids but also mechanomorphic, zoomorphic, minimalist), enormous efforts by large teams of developers and designers are put into building social robots like “Geminoid^1^” or “Sophia^2^”, which resemble their human counterparts as much as possible. Similarly, reports on robots often imply a competition to humans, with the final goal of robots acting fully human-like. For example, in a recent documentary^3^, the awarded computer scientist Bernhard Schölkopf compared self-learning robots to small children. While he still sees humans ahead, he assumes that 30 years later, people will no more be able to differentiate between a human and a robot. Considering these developments, one may get the impression that sooner or later humans and robots will interact with each other as social agents on one level, without much reflection about “being born” robot or human. Though not always explicitly communicated, the intense endeavors to create ever more human-like systems seem to suggest that missing acceptance, trust, and other current problems in human-robot interaction (HRI) can be resolved by creating the ideal system, being on par with humans.

The present research wants to challenge this view. Independent of the technical possibilities and limitations, our paper takes a more philosophical stance toward the role of robots and explores their attributed potential to become human-like by means of a thought experiment. How humans think about technology may affect acceptance, liking, usage behavior, and other facets of user experience (UX). In order to design robots with a particular intended impression on humans, as required in many application areas (e.g., care, service domains, industry settings), HRI research needs knowledge about human perceptions of robots on a meta-level such as “Can robots have feelings?” or “Can robots reflect about themselves?.” Thus, understanding human's mental models of robots forms an important basis for adequate design goals. Of course, a basis of trust and acceptance is at the heart of effective HRI. However, we put into question whether convincing humans to accept robots as a counterpart by simulating human appearance and performance as much as possible is the most promising way, and adequately fits into how humans think about robots in a conceptual sense. As one step to a better understanding of humans' mental models of robots and their self, we analyze whether in people's minds, a robot's perceived humanness depends on its similarity to human performance and appearance, or whether this is more a question of mental categorization. More specifically, we explore what might differentiate a robot with full human abilities and body parts from original humans (and vice versa).

Altogether, our research wants to shed light on how humans think about robots, and in a next step, use such insights as a more profound basis for adequate design goals. If humans will always consider robots as being fundamentally different from their own species, instead of designing most human-like robots in order to reach acceptance, it might be more promising to understand robots as an own “species” and underline their specific characteristics and benefits. In this sense, the present study may form a basis to rethink the (implicitly or explicitly underlying) design ideal of most possible human similarity, which is nowadays present in many designs of robot. Instead, our research could encourage an alternative design ideal, featuring characteristics that make it easy for humans to accept and like robots, but at the same time respecting its original nature as a technical, non-human entity. As other researchers already emphasized, identifying the whole set of factors that may affect a robot's perceived human-likeness is a complex endeavor, and anthropomorphism appears as a multidimensional phenomenon (Złotowski et al., 2014). We complement these studies by a meta perspective of studying humans' mental models and explore how humans think about robots as such, and whether, it would be possible for a robot to be regarded as on par with humans, technical limitations left aside. More specifically, referring to psychological research and biases such as the anchoring effect (for a literature review see Furnham and Boo, 2011), we assume that humans' critical reactions toward technology are not arbitrary but follow a systematic in which the starting category (e.g., human, robot) serves as an anchor for all following judgments and can hardly be overcome, regardless of an entity's later performance or characteristics. In this case, an originally non-human entity could hardly be perceived as human, even if it shares a wide amount of features with an originally human entity.

In the remainder of this paper, we present a study paradigm that simulates this effect on an abstract level with contributions in various directions. Understanding, according to human's mental models, what degree of human-likeness robots can reach in principle, can have substantial influence on our expectations toward robots as a species, on the potential tasks we will hand over to robots and on the rules and policies they have to be designed by. How human see robots is deciding for how they treat robots and which roles robots can take in a society. As described by Veruggio (2006) one possible perspective is “Robots are nothing but machines,” meaning that no matter how sophisticated or helpful robots can become, they will always be mere machines. In this view, all characteristics of a robot reflect the mechanisms and algorithms implemented by its designer and can never surpass them. The development of consciousness or even free will is impossible in this view. An alternative perspective described by Veruggio (2006) is “Robots as a new species,” which suggests that robots have autonomy and (self-) consciousness and may possibly outperform us in many ways, including the areas of intellectuality and morality (Storrs Hall, 2011). The question of a robot's *self* will also influence the acceptance and role of robots in societal systems, such as job hierarchies or other social contexts. It is therefore a decisive question for our relationship with robots in the future and the research agenda in HRI. Before presenting our study design and its rationales in detail, we discuss related work from different disciplines and research communities. When exploring the issue whether robots can (in principle) be perceived as human, a plethora of concepts come to mind which could play a role for the recognition of robots as being on par. Though we cannot discuss all these in detail, the following sections pick up central concepts and considerations from HRI, human-computer interaction (HCI), and other relevant disciplines such as philosophy and psychology.

## Related Work

### Anthropomorphism and Perceptions of Equivalency Between Humans and Technology

Within and aside from the particular domain of robots, various studies explored perceptions of equivalency between humans and technology, how people construct the difference between humans and machines, ascribed social qualities (e.g., Collins, 1993; Brooks, 2003; Kahn et al., 2006, 2012; Turkle, 2011), as well as attribution of mind. For example, Xu and Sar (2018) explored perceived differences between machines and humans along dimensions of mind perception, namely, experience and agency. They found that people see humans as superior to machines in both dimensions, however, machines in human-resemblance were perceived highest in both dimensions than other types of machines. Martini et al. (2016) explored how physically human an agent needs to appear before intentionality is bestowed onto it. To this aim, they compared images of more or less mechanistic vs. humanoid robots and studied mind attribution as dependent variable. Altogether, their findings showed that before reaching a certain threshold, human-like appearance alone does not increase mind attribution which may suggest “that agents need to be classified as having a mind first before the addition of more human-like features significantly increases the degree to which mind is attributed to that agent” (Martini et al., 2016, p. 1). Other studies explored the effect of particular design characteristics on perceived humanness of digital agents and robots, such as, for example, the effect of typefaces (Candello et al., 2017) or conversational cues (Go and Sundar, 2019) in the domain of chatbots.

Moreover, as a basic requirement for effective HCI, the question which design characteristics make users accept and engage in interaction with social technology has been a key interest of research for already over a decade. In the domain of robots, as being particularly keen to make systems appear as human-like, various studies explored how humans think about robots in (formerly) human roles such as medical staff or social companions (e.g., Kiesler and Goetz, 2002; Ljungblad et al., 2012) and the potential and consequences of anthropomorphic design (e.g., Osawa et al., 2007; Hegel et al., 2008). For example, Parise et al. (1996) found participants to be more willing to cooperate with a computer social agent who looked like a person than with two lovable dog computer agents (Parise et al., 1996). In general, a technology's ascribed humanness and subfacets thereof are components in many user studies in the context of social robots and social technology in general. For instance, Rösner et al. (2011) studied the perceived intentionality that users ascribed to the system during the course of interaction. Carpinella et al. (2017) developed a scale to measure peoples' perceptions of robotic social attributes and identified three main factors, labeled warmth, competence, and discomfort. Krüger et al. (2016) focused on anthropomorphic ascriptions of human-like mental states (e.g., motives, wishes, aims, and feelings) in the context of companion systems. They assumed such ascriptions to be motivated by a wish to turn the technology into a potential relational partner. One interesting focus of their study are user impressions regarding the technology's capabilities of the system, varying between impressive and frightening. While some users were positively impressed, others did not appraise the experienced human-like characteristics as generally positive: For them, a system which gives the impression of a machine but shows unexpected humanly performance seems scary, evoking feelings of discomfort, uncertainty and uneasy skepticism, also related to the ascription of the ability to abuse confidence to the system. Such individual differences between user perceptions could also be related to psychological traits such as individual differences in anthropomorphism. As revealed by Waytz et al. (2010), individual anthropomorphism (i.e., the tendency to attribute human-like attributes to non-human agents) also predicts the degree of moral care and concern afforded to an agent, the amount of responsibility and trust placed on an agent, and the extent to which an agent serves as a source of social influence on the self. In their study, they surveyed ratings of trust for human vs. technological agents for different tasks such as to predict heart attack risk, detect when a person is lying, determine the best college football team in the country, or select individuals to admit to a university. It showed that participants with a stronger tendency to anthropomorphize non-human agents also stated higher ratings of trust in technological agents for important decisions. Thus, in sum, numerous studies already demonstrated the general relevance of ascribed social and human-like qualities of technology for user behavior, experience and acceptance, whereby several studies imply a positive correlation between anthropomorphic technology design and/or individual anthropomorphism and trust in technological agents.

### More Complex Quality Ascriptions: Intelligence and Self

Apart from looks and basic behavior which surely will—sooner or later—reach a sufficient level of sophistication to be human-like, there are other concepts harder to grasp. In particular, concepts such as self-consciousness, the self, or even intelligence with all its facets are hard to define and even harder to measure even in humans. It has become a tradition in the field of artificial intelligence (AI) that specific capabilities once thought of as signifying intelligence are considered non-intelligent once they have been achieved algorithmically. This happened to playing Chess or Go, to face recognition and to emotion detection, to just name a few. Once a machine has successfully solved these tasks, they are suddenly not considered truly intelligent anymore, and a new domain such as playing football is declared as “the true final frontier for robotic artificial intelligence^4^.” This in turn makes true intelligence a moving target and notoriously hard to define. Apparently, our judgment associates this term with humans as a species (or at least living beings). We always seem to find counter-arguments and claim that the new capability is not true intelligence because there's something else missing. Thus, in order to further explore perceptions of equivalency between humans and technology, a critical question is what is *this something else*: So far, research has failed to provide it as a building block of intelligent systems. Following the logic above, it seems that what is missing is not something a scientist or an engineer could develop. Each new component we add to a system can in itself only be implemented algorithmically, and hence not provide true intelligence. Just as in Gestalt Psychology, it seems that the whole is more than the sum of its parts when it comes to humanness. The very concept of humanness, or a self, is hard to grasp or define, and hence invites investigation. The problem becomes even more complicated because already established methods of measurements seem to be unsuitable when it comes to robots. For example, a popular assumption for the presence of self-consciousness is the ability to recognize oneself in a mirror (Gallup, 1970). While some animals like chimpanzees are capable to learn and pass this mirror test, others are not. When it comes to robots, it would be a relatively easy task to implement the necessary features to allow a robot to pass this test. In fact, Haikonen (2007) already showed that a very simple machinery is able to pass the test and argues that the mirror test is unsuitable for robots and we need other methods of measuring self-consciousness. The problem with self-consciousness is characteristic for many related problems. The whole domain of phenomenological consciousness (e.g., what the color red looks like, what an apple tastes like) is difficult to be explained materialistically and likewise difficult to measure (Levine, 1983; Chalmers, 1995). Since it is difficult to measure, it is also difficult to prove the existence of this construct (e.g., the “qualia”). This leads to the situation that we cannot even show that other humans actually have a (phenomenological) consciousness—we rather assume the existence because we are conscious ourselves. The same holds true for robots: we cannot show that robots have a consciousness, but in contrast to humans, we have no basis to assume one. At least in our perception, this leaves robots with no real chance of being on par.

### Robots and the Self

While the word *self* is a commonly used term, the underlying concept is scientifically difficult to grasp and not yet fully understood (Damasio, 2012; Gallagher, 2013). Neisser (1988) argues that the self consists of several sub facets, which in interaction form one's self. In his analysis, he identified five different facets that can essentially be seen as different selves, because they differ in origin and developmental histories:

1. **Ecological Self**. The self in its immediate physical environment.
2. **Interpersonal Self**. Defined by engagement in human interchange.
3. **Extended Self**. Based on personal memories and anticipations.
4. **Private Self**. Based on the exclusiveness of specific experiences.
5. **Conceptual Self (or self-concept)**. Shaped by the mental representation of concepts, in which it is embedded (e.g., roles or metaphysical concepts).

If we follow this type of categorization, we have multiple starting points to create and implement a self in robots. Chella et al. (2008) distinguish between first order perception, e.g., the perception of the outer world, and higher order perception, which is the perception of the inner world of the robot. They argue that self-consciousness is based on the latter and therefore, giving a robot the ability to perceive its inner world leads to a self-conscious robot. Novianto and Williams (2009) argue in a similar way. They see a link between the concept of self-awareness and the ability of the robot to direct attention toward their own mental state. Following this line of thought, Gorbenko et al. (2012) propose a model that generates robot-specific internal states. In line with Novianto and Williams (2009), they argue that a robot needs a capability to attend to its internal states to be self-aware. They provide a list of concepts, which can constitute a robot's internal state, including emotion, belief, desire, intention, as well as sensation, perception, action, planning, and thought. While those concepts are also present in humans, they emphasize that developers should not mimic the internal state of humans but should rather focus on robot-specific needs. Finally, Pointeau and Dominey (2017) explore the role of memory for the robot self. They build on the arguments of Neisser (1988), who emphasizes the ecological nature of the self and the development over time. Pointeau and Dominey (2017) take up this thesis and argue that it should be possible for a robot to build up its own autobiographical memory through engagement in the physical and social world and, as a result, develop aspects of a self in its cognitive system.

Altogether, the self can be viewed as an umbrella term, containing several facets and providing different ways to artificially create it. At least in theory. The question remains if humans will grant robots their own self or if they will deny it for whatever reason. Below, we will use a working definition for the concept of the self, seeing it as the original identifying essence of an individual.

### Research Motivation

Our study aimed to find out whether, according to humans' mental models, it would ever be possible to create a robot which can be perceived as equal to humans. We assume that the issue here is not so much a question of technical advancements but more one of psychological concepts: Humans tend to perceive themselves as being special in various ways, e.g., being the “pride of creation.” Allowing another type of being to be on par with us could challenge our self-esteem and our identity. Therefore, it is plausible to deny any type of equality and emphasize the differences (e.g., “playing Go is no real intelligence because it cannot artistically play a guitar”) more than the similarities. With this in mind, we designed a study with the goal to investigate the point from which on robots would be considered human, or humans would not be considered humans anymore. More specifically: will humans evaluate equal functionalities and skills in humans and robots equally, or will they evaluate them differently? Will the self, as a central construct related to identity and personality remain unaffected or will it dwindle away in the process?

To answer these questions, we set up an experimental study of humans' mental models of robots based on fictional transitions from human-to-robot and robot-to-human.

### Study Paradigm and Methods

Our study paradigm realized two experimental conditions of fictional transitions, namely, a human-to-robot condition and a robot-to-human condition. The transition consisted of 20 steps. After each step, the participants gave a rating about the depicted entity.

In the human-to-robot condition, the participants started with a complete human, which went through a procedure of 20 subsequent steps, whereby in each step, one part or area of the human (e.g., legs, heart, emotions, logical thinking) was replaced with robotic parts providing equal functionalities. After the twentieth step, the human was fully replaced with robotic parts. After each step, the participants rated the remaining humanness (and the consequential robotness) and remaining self of the depicted entity (i.e., human-robot-mixture). Thus, the study of ratings along the transition can provide insights into potential critical turning points and the question, whether robots can ever be perceived as human-like, if they fulfill all objective requirements.

In the robot-to-human condition, the procedure was the same, except for the starting point: Here, participants were confronted with a complete robot of human proportions, which was successively replaced with human parts. After each step, the participants rated the remaining robotness (and the consequential humanness) and remaining self of the depicted entity (i.e., human-robot-mixture).

In order to explore the assumed anchoring effect (i.e., a high impact of the starting entity on the rated humanness or robotness), it was necessary to have a fixed set of replacements, whereby the perceived humanness/robotness can be viewed from two directions (human-to-robot, robot-to-human). Therefore, the study design was balanced (starting the transition with a full human vs. full robot), but the order of body parts replaced differed between the human-to-robot-transition (starting with legs, mouth, rationality…and finally arms) and the robot-to-human-transition (starting with arms, ears, emotions…and finally legs). This study design provides comparable entities in both experimental conditions. For example, regarding the body parts, the resulting entity in the human-to-robot condition after two exchange steps (i.e., robotic legs, robotic mouth, all other parts human) is comparable to that in the robot-to-human condition after eighteen steps. For each of these points of comparable entities and specific combinations of body parts, we could compare the ratings of the perceived humanness/robotness depending on the starting point of the transition (human, robot) and the experimental condition and test the assumed anchoring effect. If we had used the same order of replacements (e.g., starting with legs in both conditions) this analysis would not have been possible, because not only the starting point of the transition, but also the combination of body parts would have been different in the two conditions.

To assure transitions and changes of body parts of comparable significance in both directions we performed a prior workshop. The aim of the workshop was to identify relevant parts of humans/robots (e.g., legs, eyes, memory), to rate these parts regarding their significance for humanness/robotness and the self, and to identify a sensible order of these parts to create transitions of comparable significance. For example, one might argue that memory is more relevant for the self than legs.

**Figure 1:**
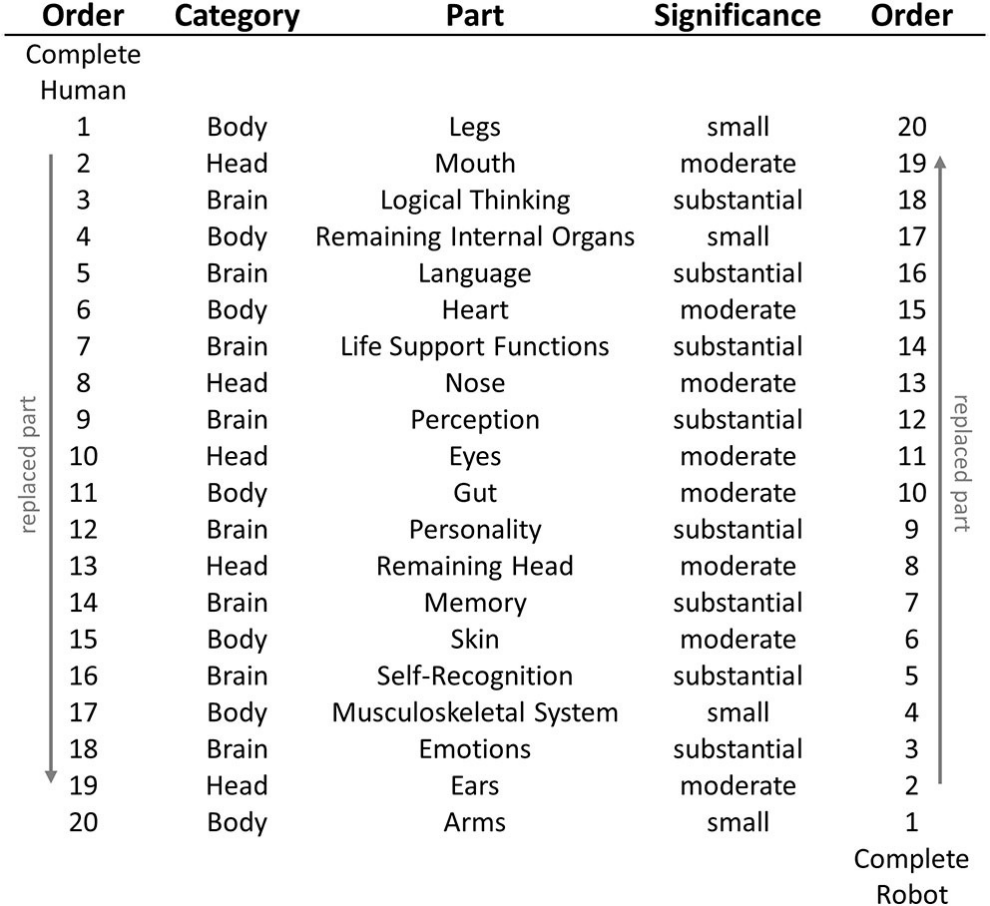
Parts replaced in each step of the procedure, their category and significance for humanness/robotness and self and order number in the respective condition (left: starting with a complete human, right: starting with a complete robot).

The workshop was performed with three participants with background in HCI, HRI, and psychology. A brainstorming session led to a list of exchangeable human/robotic body parts, aiming at a collection of all potentially exchangeable parts, i.e., a full transition. The participants then discussed how significant this specific part was for an entity's self and its belonging to its “species” (here: human or robot). The workshop was organized as a group discussion, leading to a joint group rating. For each part, the participants gave a unified rating of significance (small, moderate, or substantial). For example, the group discussed how significant it was for the remaining human self if a human had its legs replaced by robotic legs (rated as being of small significance), compared to a change of the eyes (rated as moderate) or the language (rated as substantial). Based on the participants' subjective ratings and a detailed analysis after the workshop, we selected 20 definable parts for our study which can be categorized in three clusters: (1) parts of the brain and attributed functionalities (e.g., emotions, language center), (2) parts of the head (e.g., eyes, mouth), and (3) parts of the remaining body (e.g., heart, musculoskeletal system). For a detailed list of the parts and their attributed significance, see Figure 1.

### Participants

Four hundred eighty-one participants (55.5% women, 34.5% men, 10% gave no information) took part in the main study, the age range was 17–74 years (M = 25.9, SD = 9.76). The participants were recruited via mailing lists and were incentivized by giving the chance of winning amazon coupons. The participants were predominantly students or came from an academic environment. The study was implemented as an online survey with a mean duration of 24 minutes (min = 8, max = 80, SD = 12.6) and consisted of four parts.

### Procedure and Measures

In the first part and the introduction of the survey, the participants were told to assume a technology of being capable to virtually replace any human part with a robotic part and vice versa. This scenario touches upon current design trends and the aforementioned robots like “Sophia” or “Geminoid,” implying the notion to make technology more “perfect,” by adding ever-new human-like features (e.g., simulating human voice and dialogue, human-like motion, human-like facial appearance). The participants were informed that they should ignore all technological issues related to replacing parts and should assume a fully functional replacement procedure. Then, the participants were randomly assigned to one out of the two conditions, resulting in 246 participants in the human-to-robot condition and 235 participants in the robot-to-human condition. In the human-to-robot condition, the participants started with a complete human which went through a procedure of 20 subsequent steps. In each step, one part or area of the human (starting with the legs) was replaced with robotic parts providing equal functionalities. After each step, the participants rated the remaining humanness and remaining self of the depicted entity on a scale from 0 to 100%. After the twentieth step, the human was fully replaced with robotic parts, which was also noted in the study. In the robot-to-human condition, the procedure was the same, except for the starting point: Here, the participants were confronted with a complete robot of human proportions, which was successively replaced with human parts. Thus, the instruction described the robot only vaguely and did not provide further information about its appearance, purpose or other details. As noted above, the twenty parts were replaced in inverted order between the two conditions, thereby allowing comparisons of equal human-robot-mixtures (see Figure 1). While the legs were replaced first in the condition with the human starting point, they were replaced last when starting with a robot. Note, that we cannot be sure whether all participants had the same imagination of the starting entity or the procedure of “replacing” parts. However, since we were interested in the participants' unbiased personal mental conceptions of robots and humans, we deliberately limited information about the starting entities, and rather learnt about the participants' different personal mental models from the analysis of open statements.

In the second part of the study, we asked the participants qualitative questions about the replacement process and the perceived difficulty of the evaluation tasks (ratings of humanness/robotness and remaining self). One question was whether the participants rated the completely replaced human (robot) now as completely robot-like (human-like), and if not, how the participants came to their opinion. Further questions were related to the most important part which would make a human (robot) being human-like (robot-like) and which was most important for conserving the self. We also asked whether the participants missed a crucial part in the replacement process which was not explicitly replaced. The qualitative statements were categorized based on the approach of qualitative content analysis. More specifically, the procedure followed a procedure of inductive category development, as described by Mayring (2000). Inductive category development consists of a step by step formulation of inductive categories out of the material. The material is worked through and categories are tentative and step by step deduced. In the beginning of the process, each qualitative statement might form a new category. Then, for each qualitative statement, it is checked whether this can be subsumed under one of the existing categories or whether a new category needs to be formulated. For example, regarding the question why the transformation process does not lead to a completely human-like entity in the robot-to-human condition, one statement was “It just lacks a soul,” building a first category labeled “no soul.” Another statement was “A human is more than the sum of its parts,” building another new category. Also the statement “A human is not the sum of its parts” was subsumed under this same category, labeled “Human is greater than the sum of its parts.” Within feedback loops, categories are revised and eventually reduced to main categories and checked in respect to reliability. The category development was performed by an independent rater (a psychologist, trained in qualitative data analysis). Then, a second rater (also psychologist and trained in qualitative content analysis) categorized the open field responses based on the developed categorization scheme. The interrater agreement was satisfactory, with Cohens Kappa values between 0.78 and 0.86 for the different questions. Finally, we surveyed ratings of task difficulty. Participants stated how difficult it was for them to rate the remaining ratio of self and humanness/robotness for the different human-robot-mixtures on a 7-point-scale ranging from easy (=1) to difficult (=7).

In the third part, we asked additional qualitative, open questions related to participants' attitude and understanding of the relevant concepts (e.g., the self). We asked the participants, how they would define the self, where they would locate the self (if anywhere), and whether they thought that robots were capable of-−1 day—developing a self. Furthermore, we asked the participants about their beliefs in respect to a soul, to god, and in generally spiritual or metaphysical levels.

The fourth and last part of the survey consisted of demographic questions, such as age, gender, and educational background.

## Results

### Attributions of Remaining Self and Humanness/Robotness for the Two Transitions (Human-to-Robot, Robot-to-Human)

**Figure 2:**
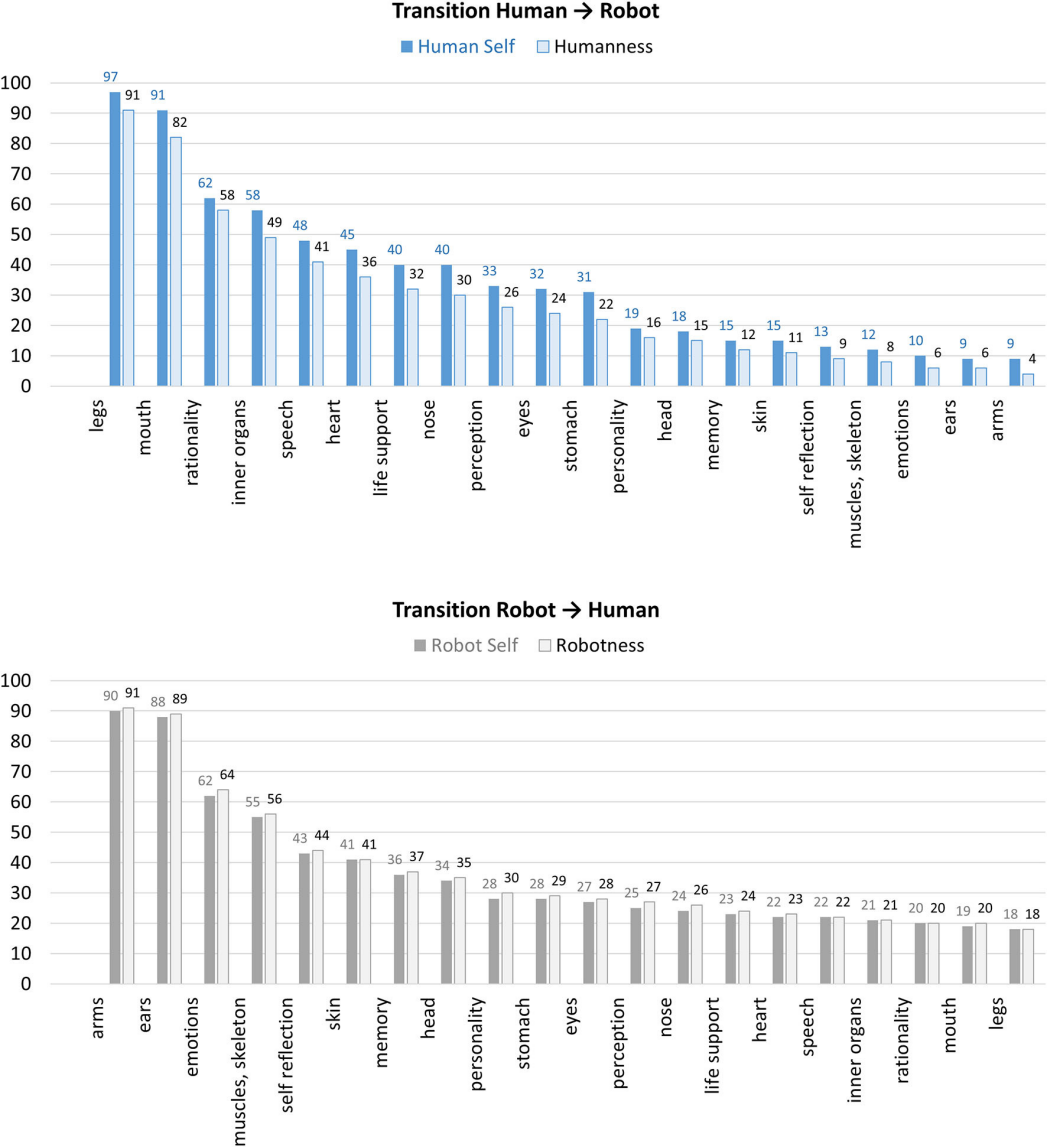
The participants' ratings of the remaining ratio of self and humanness in the human-to-robot condition (top) and ratings of the remaining ratio of self and robotness in the robot-to-human condition (bottom) at different points of transition.

Figure 2 shows the participants' ratings of the remaining ratio of self at different points of transition for the two experimental conditions (human-to-robot, robot-to-human). In addition, Figure 2 depicts the participants' ratings of the remaining ratio of humanness (in the human-to-robot condition) or the remaining ratio of robotness (in the robot-to-human condition). It shows that for both measures, the formerly 100% robot retains a higher degree of self/robotness at the end of the transition than the formerly 100% human does for self/humanness, respectively. After the full transition and exchange of all specified parts, the former human is only attributed 4% humanness and 9% self left. In contrast, after the objectively same transition and exchange of the same parts, the robot is still attributed 18% robotness and 18% self left.

**Figure 3:**
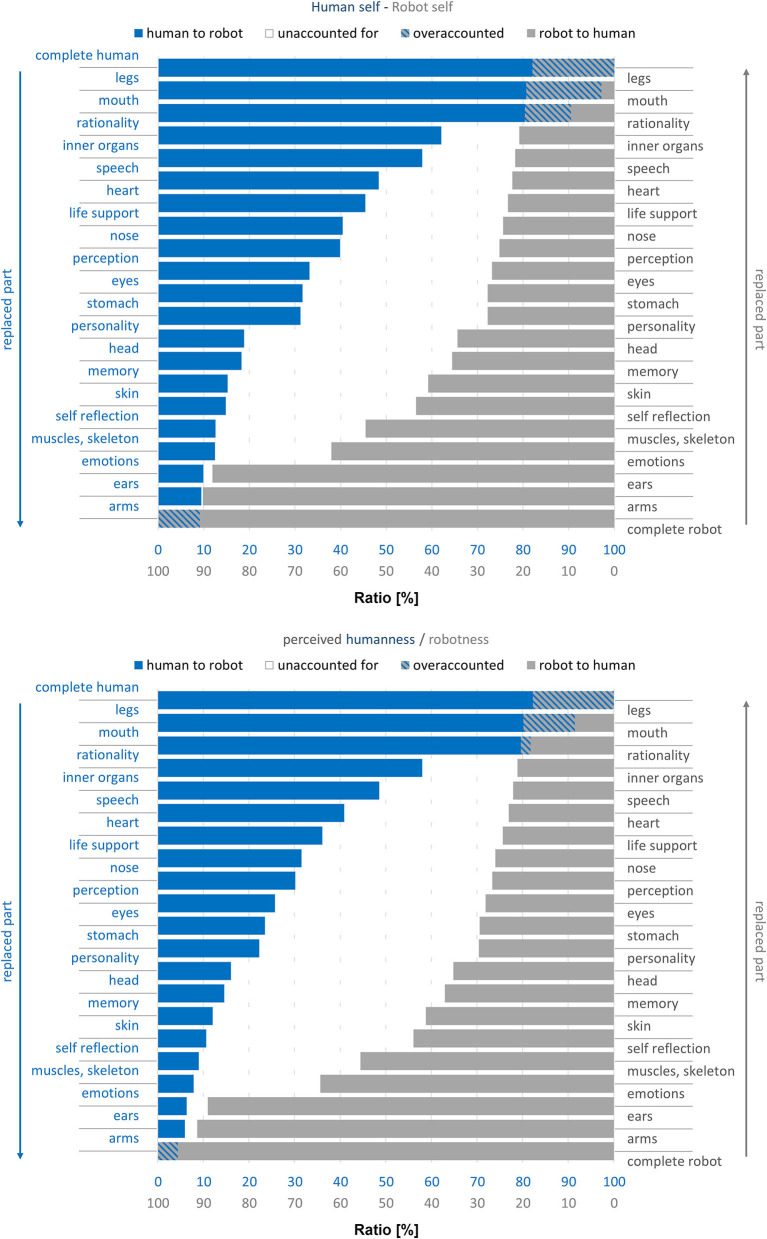
Combined findings of the two experimental conditions for ratings of remaining self (top) and remaining humanness vs. robotness (bottom).

For an additional analysis, Figure 3 displays the combined findings of the two experimental conditions in one diagram. The diagram shows the transformation process from both sides, starting with a complete human (left side, from top to bottom) and a complete robot (right side, from bottom to top). The x-axis represents the degree of remaining self or perceived humanness/robotness, respectively. Thus, a fully blue bar indicates a remaining self or humanness rating of 100% if starting with a complete human. A completely vanished blue bar (0%) indicates a remaining self or humanness rating of 0%. The same applies with mirrored axes for gray bars when starting with a complete robot. Each bar represents the mean evaluation of remaining self or humanness/robotness after each step in the replacement process. With this type of visualization, we can compare identical human-robot-mixtures. For example, the second bar starting from top shows the data for a human with replaced legs (blue bar) and that for a robot with everything replaced but the legs (gray bar). The middle area highlights the unspecified gap between the two transitions, showing that ratings of robotness and humanness for an identical human-robot-mixture do not add up to 100%. Indirectly, this speaks against the mental model of a simple one-dimensional continuum of human- vs. robotness, where one would have findings such as “A former robot that got human arms and ears is now 10% human and 90% robotic.”

Participants' ratings of how difficult (1 = easy, 7 = difficult) it was to rate the remaining ratio of self and humanness or robotness showed mean values above the scale midpoint of 4 for all surveyed difficulty ratings. More specifically, the participants' mean difficulty rating was M = 4.34 (SD = 1.78, *t*_(240)_ = 2.94, *p* < 0.01) for the remaining ratio of humanness/robotness and M = 4.19 (SD = 1.91, *t*_(240)_ = 1.55, *p* > 0.05) for the ratings about the remaining ratio of self in the human-to-robot condition. In the robot-to-human condition, the difficulty ratings were M = 4.48 (SD = 1.93, *t*_(224)_ = 3.73, *p* < 0.001) for humanness/robotness and M = 4.28 (SD = 1.96, *t*_(224)_ = 2.11, *p* < 0.05) regarding the remaining ratio of self. As shown by the calculated one sample *t*-tests, for three of the four surveyed difficulty ratings, the difference to the scale midpoint was significant, implying that the task was rather difficult than easy for the participants. In addition, open answers indicated that the participants experienced the study as quite sophisticated but also lots of fun and inspiring since it activated interesting questions one had not considered beforehand.

### Reasons Given for Attributed Self and Humanness vs. Robotness

After the participants had made their ratings of remaining self and humanness/robotness, they were asked to further explain their attributions by qualitative statements, which were categorized as described above. The first question was “If now that all parts have been exchanged, you still think the human/robot is not yet fully robot-like/human-like—why? Please state your reasons!.” A first insight was a significant difference between the ratio of the participants who agreed and answered this question between the two experimental conditions: While only 29 out of the 246 participants (12%) in the human-to-robot condition answered this question, 81 of the 235 participants (34%) did so in the robot-to-human condition (χ^2^(1) = 35.05, *p* < 0.001). Thus, a higher ratio of participants found that a former robot with human body parts is not fully human-like, whereas less participants saw a former human with robotic body parts as not fully robot-like. In other words, humanness seems harder to gain than robotness. Among the stated reasons for the robot not having become human-like, the most frequent category of mentions (32%) concerned the (unnatural) process of production/development. For example, one person gave the reason “Because it has not developed naturally”. About 14% argued that no matter how many parts are exchanged, the individual keeps its original entity. A sample statement in this category was “It is a machine and it remains a machine—no matter what you change about the material.” Tables 1, 2 show the categorized reasons and sample statements for the two experimental conditions.

**Table 1:** The participants' reasons why the transformation process does not lead to a completely robot-like entity in the human-to-robot condition.

Question: If you think the human is still—after replacing all parts—not completely robotlike, why is that?		
**Category**	**Sample-item**	**Occurence**
Self/Emotions are human	“*The Self is still a human. There wasn't anything new created.”*	36%
Humanness is eternal	“*After all, the starting point was human.”*	23%
Soul is human	“*He is barely robotlike, but the soul still exists.”*	18%
Solely optic differences	“*Apart from optical aspects there are no differences. Optics do not define humans.”*	9%
Missing organic basis	“*The FOXP3-Gen makes a human being human. Replace it and the human is gone.”*	9%
Human is both human and robotlike	“*He is both human- and robotlike.”*	5%

**Table 2:** The participants' reasons why the transformation process does not lead to a completely human-like entity in the robot-to-human condition.

Question: If you think the robot is still—after replacing all parts—not completely humanlike, why is that?		
**Category**	**Sample-item**	**Occurence**
No natural origin	“*Because it has not developed naturally.”*	32%
Other reason	“*I have problems with the separation process.”*	16%
The essence survives the transformation	“*He is a machine and no matter what is changed he remains a machine.”*	14%
Human is greater than the sum of its parts	“*A human is more than the sum of its parts.”*	12%
Missing development	“*He has not passed through a growth process.”*	10%
No soul	“*It just lacks a soul:)”*	9%
Missing memories	“*He has no memories.”*	4%
Organic disparities	“*After all, there is a lot of technology involved.”*	4%

As a next question, we asked the participants whether they believed that robots could develop a self. Table 3 displays the stated reasons why robots can or cannot develop a self, categorized for yes- and no-answers. While 40% were sure that robots can develop a self, about the same ratio of participants (42%) was sure they cannot. Fifteen percentage % were undecided, 3% said “rather no,” and 1% “rather yes.” In addition, the participants further qualified their rating by open statements. While some participants argue that technological advance will make this possible, and that humans are “only bio-machines as well,” others see this as impossible, since a self cannot be programmed or added artificially.

**Table 3:** Reasons why robots can or cannot develop a self.

Question: Please back up your opinion: Why/why not?		
**Yes/No**	**Category**	**Occurence**
Yes	By means of technological progress	19%
	Machines are capable of learning	11%
	Humans are only biological machines	10%
	Machines are only programmed	22%
	Only machinelike	20%
	Problem too complex	5%
No	Not possible *post-hoc*	4%
	Missing emotions	4%
	Missing soul	3%
	Missing personality	2%

### The Essentials of the Self

**Figure 4:**
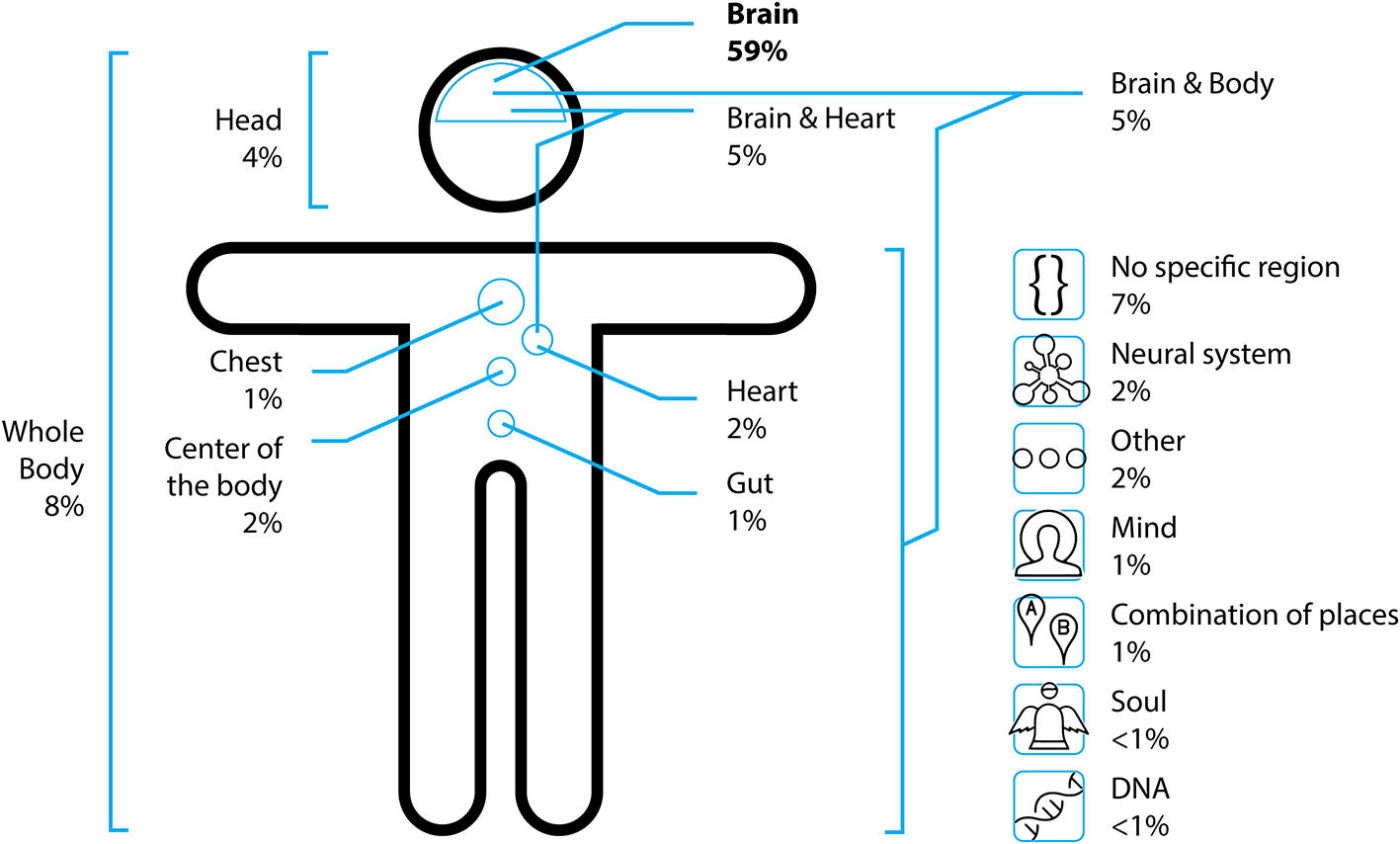
The participants' assumptions about the physical location of the self.

In order to get a closer idea of the participants' mental model of “the self,” we asked them where they would assume the self (e.g., in a particular body region). As shown in Figure 4, the clearly most frequent answer was the brain (59%), followed by “in the whole body” with 8%. Other answer clusters included combinations of body parts or more vague concepts like “no specific region” and were each mentioned with a frequency of 7% or below.

Finally, we asked the participants to pick (in a drop-down menu) the part that according to their view is the most essential for attaining the self, also providing the option to differentiate between different brain parts related to particular functions. Table 4 shows the participants' ratings for the different options provided. As it can be seen, most participants see the attainment of self-related to the personality brain part. Other frequent mentions refer to the brain part accountable for memories or the brain part referring to reflections about oneself.

**Table 4:** Most important parts (out of the 20 included in the present study) to conserve the self.

Question: Which part is most essential to conserve the self?	
**Category**	**Occurence**
Brain part: Personality	51%
Brain part: Memory	15%
Brain part: Self-recognition	12%
Brain part: Emotions	9%
Brain part: Life support functions	4%
Brain part: Perception	3%
Brain part: Logical thinking	3%
Brain part: Language	1%
Arms	<1%
Musculoskeletal System	<1%
Parts of the Head	<1%
Heart	<1%
Mouth	<1%

## Discussion

Our research used fictional transitions from human-to-robot and robot-to-human to gain insight into humans' mental models of robots and their self. In each step of the presented transitions, one part or area of the human was replaced with robotic parts providing equal functionalities and vice versa and participants rated the remaining humanness (or robotness) and remaining self of the depicted entity. Based on the combined analysis of our quantitative and qualitative data, the following paragraphs highlight three central issues and possible interpretations, i.e., (1) an anchoring effect, where the starting category is decisive for attributed humanness or robotness, (2) humanness appearing as a more sensible attribute than robotness, and (3) a more complex relationship between humanness and robotness than a one-dimensional continuum.

Participants' ratings of the remaining degree of self and humanness/robotness for the different human-robot-mixtures showed that the starting category (e.g., human, robot) was decisive for all subsequent judgments and can hardly be overcome, as also suggested by the psychological anchoring bias (Furnham and Boo, 2011). Even if all body parts had been exchanged, a former robot was not perceived as totally human-like and a former human not as totally robot-like, implying that the starting entity always remains relevant. At the same time, the origin as human or robot cannot fully protect against (partly) losing one's original self. In fact, in both experimental conditions the exchange of already a few parts were associated with quick losses of the former self. For example, the exchange of four parts, implied already losing about half of one's former identity (i.e., being now 56% instead of 100% robotic or 49% instead of 100% human).

The comparative analyses of ratings in the two experimental conditions suggest humanness as a more sensible attribute than robotness. The formerly 100% robot retains a higher degree of self/robotness at the end of the transition than the formerly 100% human does for self/humanness, respectively. In other words, the rate at which humans lose their humanness is higher than the rate at which robots lose their robotness. Moreover, in the then following question, a higher ratio of participants found that a former robot with human body parts is not fully human-like, whereas less participants saw a former human with robotic body parts as not fully robot-like, suggesting humanness as the harder to gain attribute. A possible interpretation is that humanness is considered more fragile or volatile, one might say “precious,” than robotness. For example, even exchanging two parts leads to a dramatic loss in humanness and after exchanging all specified 20 parts, virtually no (only 4%) humanness is left. At the same time, humanness cannot fully be created artificially. Even if a former robot has all parts exchanged, so that it literally consists of the same features as a human does, it is still attributed 18% robotness—implying that it is no full human yet. From a neutral point of view, assuming that robotness or humanness are just two attributes and none of the two is more desirable than the other, one could also state that robotness is more robust. If you are “born” as a robot, some part of you always remains robotic, even if from a feature perspective you are no longer discernable from a human being. In contrast, humanness appeared as a more special and sensible attribute which an individual can more easily loose.

Finally, another central insight was that a simple one-dimensional continuum between human and robot, as suggested by our thought experiment, does not reflect how humans reflect about robots and differences to their own species. This followed from the combined findings of the two experimental conditions in one diagram as depicted in Figure 3, especially the middle area of unspecified gaps between the two transitions. Obviously, the perceived degree of humanness and robotness do not add up to 100% for any given number of exchanged parts. If in a humans' mental model for each point of transition there was a fixed ratio of humanness and robotness, the two corresponding bars for different points of transition within the two experimental conditions would add up to 100%. However, the middle area shows that there are large ratios unaccounted for, also implying that non-robotness does not automatically imply humanness (and vice versa). It shows that the thought experiment, imposing a simple one-dimensional continuum between human and robot, does not accord to participants' mental models of human and robots. Instead, this hints at a mental model of humanness and robotness as rather vague attributes which do not necessarily add up to 100%. However, it is not clear with what else the remaining “empty” ratio is filled. Altogether, the question of assigning humanness or robotness seems more complex than counting exchanged body parts. In line with this, participants rated the attribution tasks as rather difficult than easy. The complexity of the issue was further reflected in participants' diverse statements about whether robots can develop a self, resulting in a variety of reasons for and against. Referring to the different views on robots as introduced above (Veruggio, 2006), many of the participants' statements could be broadly allocated to the two extremes of “robots are nothing but machines,” seeing no chance for robots to go beyond the machine level vs. “robots as a new species,” even seeing a chance that robots may outperform humans in valued areas such as intellectuality and morality. In parallel to these two contradicting positions, the participants in our study also provided arguments in both directions: Among the reasons given for a robot having a self (or not), a considerable number of the participants argued that due to its artificial process of production/development, a robot could never have a self. This is in line with the “nothing but machines” position. The sample statement “It is a machine and it remains a machine—no matter what you change about the material” perfectly summarized this. It might be that these participants see something “holy” in the human species which can never be overcome and not be ruled out by any pragmatic argumentation about an individual's objective abilities. On the other hand, other participants applied the same argument for humans, labeling humans as “bio-machines,” and thus seeing no fundamental difference between humans and robots and their chances of having a self. Those participants held a pragmatic view, deciding the question about having a self-dependent on one's abilities, and if technological advance should equip robots with self-awareness abilities, they saw no barrier to attribute robots a self.

In sum, the combined analyses of our quantitative and qualitative data therefore suggests that the starting category is decisive for an entity's attributed humanness or robotness, whereby humanness appears as a more sensible attribute than robotness, and the relationship between humanness and robotness seems more complex than a one-dimensional continuum. Transferred to the praxis of robot design, this creates a new perspective on the design and development of robots oriented on human ideals. Even if 1 day, there should be no more discernable difference in appearance and performance, humans still will probably not consider robots as being on par with humans. In our study, humanness appeared to be a sensible characteristic, and participants provided various reasons why in their view, a former robot with human body parts was still not completely humanlike. The explanations ranged from missing memories, the missing growth process or natural origin, lacking a soul, or the impression that “a human is more than the sum of its parts.” There might be some implicitly ascribed properties that cannot be traced to specific parts, leading us back to the Gestalt concept and the secret of what exactly makes something more than the sum of its parts.

## Limitations

As a basic general limitation, the present research only referred to singular aspects of humans' mental models of robots, centering around fictional transitions between human and robot and exchanging body parts, as well as participants' ideas about a robot's self. It can be questioned to what degree the present transformation paradigm can actually assess people's understanding and ascription of humanness to robots, and vice versa. The paradigm implicitly defines humanness as a combination of parts, and forces people to evaluate this combination of parts, which of course neglects notions of humanness and self being constructed within interactions with others. This, however, is also what design approaches implicitly suggest that aim to build human-like robots by simulating their appearance and abilities. Thus, while in general, mere body parts can surely not be seen as sufficiently indicative of humanness or robotness, we applied this limited view in context of the present thought element to explore humans' reactions and the effects of the starting category.

One aspect which could have had a great impact on the participants' ratings on humanness/robotness was the number of steps involved in the transformation process. Our aim was to cover most functions and facets of human biology and psychology, which resulted in 20 distinctive parts. However, this rather high number could have led to a data artifact in the sense, that the participants would remove a huge portion of humanness/robotness after the first replacements, leaving little for the later steps. On the other hand, when asked if specific parts were missing in the replacement process, 8.5% out of all participants stated the process was lacking a part, mentioning reproductive organs most frequently.

Another issue comes along with the specification and functionality of the brain parts. For instance, we discovered in literature research and prior research that personality is a crucial aspect for identity and significantly shapes the impression of a human/robot. However, there is no single distinguishable part in the brain that is exclusively accountable for personality. Nonetheless, we needed this concept in the study and the results indicate that it is the most important for the self. While laypeople are unlikely to have issues with this conceptual vagueness, experts on the field could stumble upon it.

Furthermore, we compared the loss of humanness/robotness and the self between two conditions (starting with a complete human vs. robot), having the parts replaced in reversed order. This was necessary in order to make comparisons of equal human-robot-mixtures (see Figure 3). Thereby, however, the sequence was not the same for the individual transformation processes (see Figure 2), opening a potential alternate explanation for the different development of loss of humanness/robotness. While we tried to balance the significance of the single parts across conditions, further studies should vary the replacement order.

In our paradigm, after each step the participants rated the humanness/robotness and remaining self. A decreasing rating for humanness (in the human-to-robot condition) came along with an increased robotness rating (e.g., adjusting a slider from 100% humanness to 0% humanness = 100% robotness). However, as discussed above, such a simple one-dimensional model is not reflected in participants' answers. Considering the combined findings of the different conditions (see Figure 3), the assumption that a loss in humanness necessarily leads to a gain in robotness does not hold true. Thus, while the present study design and one-dimensional measures were helpful to reveal that humans' mental models of robots are more complex (as also highlighted by the unaccounted areas in Figure 3), this approach represents a restriction at the same time. The applied one-dimensional measures cannot express participants' perspectives in full. Therefore, the ratings for humanness/robotness should possibly be split in two separate ratings and complemented by qualitative data.

Another possible limitation originates from the concept of the self. We used the self as an umbrella term in order to cover many facets of identity and aspects that makes an entity human. While we arguably achieved to cover a broad range of associations what defines a human, the participants made their ratings on possibly different assumptions. A segmentation of the self in several sub facets or replacing it with other concepts (e.g., identity) could pose an alternate option for future studies.

Finally, our participants were predominantly students with a western cultural background and socialization. Participants with another cultural background—and possibly another relationship to spirituality or materialism—could perceive the transformation process differently.

## Implications and Future Perspective

In sum, our findings suggest that according to human's mental models, an individual's origin always makes a critical difference. Even if due to technological transitions a former human and robot consist of the same parts (or vice versa), they are not attributed the same degree of humanness/robotness. However, aside from this evidence that there is some difference between humans and robots regarding the robustness of the self, our study can still not provide a clear picture of how humans see robots in general but rather underlines the complexity of the topic, including considerable interindividual differences. Even more, this suggests a further exploration of humans' mental models of robots, also aiming to identify possible underlying factors of interindividual differences such as, for example, individual anthropomorphism or spirituality. In addition, future research needs to pay attention to the consequences of one's view of robots and their self and other attributions and behavior, such as trust and willingness to interact with a robot.

In order to design robots with a particular intended impression on humans, as required in many application areas (e.g., care, service domains, industry settings), HCI research needs knowledge about human perceptions of robots on a meta-level such as “Can robots have feelings?” or “Can robots reflect about themselves?” Lacking insights of peoples” general imagination of “robots as a species” may lead to disadvantageous effects in design and marketing. To name just one example: As reported by Waytz et al. (2010), General Motors (GM) once ran an advertisement to demonstrate their commitment to manufacturing quality. The slightest glitch in production would not meet their quality standards, so the intended message. The advertisement depicted a factory line robot being fired from its job after it inadvertently dropped a screw it was designed to install in a car. In the following, the ostensibly depressed robot takes a series of low-level jobs until it becomes “distraught” enough to roll itself off a bridge. Instead of GM's manufacturing quality, the public attention rather focused on the interpretation that depression had led the easily anthropomorphized robot to commit suicide. The ad even alerted the American Foundation for Suicide Prevention being concerned about the portrait of “suicide as a viable option when someone fails or loses their job” and that “graphic, sensationalized, or romanticized descriptions of suicide deaths in any medium can contribute to suicide contagion^5^.”

Currently, research in HRI often focuses on designing robots as human-like as possible. While this approach seems promising for narrowing the gap between humans and robots at first sight, our results suggest that these endeavors might eventually be futile, and even counterproductive. The design ideal of human-likeness, which is very costly, complicated, and technically complex to implement, is not what will make robots become fully integrated entities in our society. If robots will always retain some degree of their robotness (being “the eternal robot”), it might be more promising to also design them accordingly. Instead of blurring the line between human and robot, the design of robots could instead emphasize the specific characteristics of robots as a separate species. Popular figures in Science Fiction, such as C3PO in Star Wars or Lt. Data in Star Trek show that robots with emphasized robotic properties can fulfill very useful functions in a society, partly because their robotness is emphasized instead of hidden. In a way, this makes an argument for a pluralistic society in which robots can play out their own strengths instead of having to (unsuccessfully) mimic humans.

First examples of approaches in such a direction is to explicitly focus on robot's special abilities beyond human abilities (e.g., endless patience) and to consider these as “superpowers” (Welge and Hassenzahl, 2016; Dörrenbächer et al., 2020). and colleagues. Similarly, Clark et al. (2019) refer to alternatives to most human-like design in the domain of conversational agents. Based on a qualitative study, they conclude that “Conversational agents promise conversational interaction but fail to deliver” (Clark et al., 2019, p. 1). In consequence, they suggest that “conversational agents can be inspired by human-human conversations but do not necessarily need to mimic it” and recommend to consider human-agent conversations as a new genre of interaction.

We hope that the present work might inspire more reflections in such directions and will add to a closer integration of people's mental models of robots with design ideals and their role in our society. Naturally, such studies of mental models can never be seen of ultimate validity. The present findings represent a current snapshot of the public perception of robots, which in turn will remain a moving target. More and more robots with improving capabilities entering our society will invariably lead to a stronger habituation and potentially a higher acceptance, or at least a more differentiated stance on robots. This might also include accepting their authority in areas in which they might be clearly superior (a near-term example of this being self-driving cars), or eventually also accepting social robots as another species in our society.

## Data Availability Statement

The raw data supporting the conclusions of this article will be made available by the authors, without undue reservation.

## Ethics Statement

Ethical review and approval was not required for the study on human participants in accordance with the local legislation and institutional requirements. Written informed consent from the participants' legal guardian/next of kin was not required to participate in this study in accordance with the national legislation and the institutional requirements.

## Author Contributions

DU and SD conceived and planned the study. DU carried out the study and performed the data analysis. All authors discussed the results and contributed to the final manuscript.

## Conflict of Interest

The authors declare that the research was conducted in the absence of any commercial or financial relationships that could be construed as a potential conflict of interest.

## Footnotes

^1^https://www.laurinci.com/hiroshi-ishiguro

^2^https://www.hansonrobotics.com/sophia/

^3^https://www.3sat.de/wissen/nano/190913-sendung-nano-102.html

^4^https://theconversation.com/why-football-not-chess-is-the-true-final-frontier-for-robotic-artificial-intelligence-62296

^5^http://money.cnn.com/2007/02/09/news/companies/gm_robotad/
